# Acute adrenal crisis after orthopedic surgery for pathologic fracture

**DOI:** 10.1186/1477-7819-5-27

**Published:** 2007-03-06

**Authors:** Norifumi Naka, Satoshi Takenaka, Katsuhiko Nanno, Yu Moriguchi, Bang-mi Chun, Shunji Sonoda, Nobuyuki Hashimoto, Yoshitane Tsukamoto, Nobuhito Araki

**Affiliations:** 1 Department of Orthopedic Surgery, Osaka Medical Center for Cancer and Cardiovascular Diseases, Osaka, Japan; 2 Department of Anesthesiology, Osaka Medical Center for Cancer and Cardiovascular Diseases, Osaka, Japan; 3 Department of Pathology, Osaka Medical Center for Cancer and Cardiovascular Diseases, Osaka, Japan

## Abstract

**Background:**

Adrenal crisis after surgical procedure is a rare but potentially catastrophic life-threatening event. Its manifestations, such as hypotension, tachycardia, hypoxia, and fever mimic the other more common postoperative complications. Clinical outcome is dependent upon early recognition of the condition and proper management with exogenous steroid administration.

**Case presentation:**

We report a 75-year-old man who presented with shock immediately after surgery for a femoral fracture from lung cancer metastasis. Anemia and severe hyponatremia were detected. Despite adequate fluid resuscitation, nonspecific symptoms including hypotension, tachycardia, hypoxia, fever and confusion occurred. Emergent CT revealed enlarged bilateral adrenal glands. Under the diagnosis of adrenal crisis due to metastatic infiltration of adrenal glands, the patient was treated with appropriate steroid replacement resulting in rapid improvement and recovery.

**Conclusion:**

We describe a case of adrenal crisis caused by the lack of adrenal reserve based on metastatic involvement and surgical stress, the first published case of adrenal crisis after surgery for a pathologic fracture from lung cancer metastasis. Surgeons treating pathologic fractures should be aware of this complication and familiar with its appropriate therapy because of increasing opportunity to care patients with metastatic bone tumors due to recent advances in cancer treatment.

## Background

Adrenal crisis is a potentially lethal entity that is rarely reported after surgical procedures. The clinical manifestations are nonspecific and difficult to distinguish from common symptoms such as fever, hypotension, tachycardia, and electric disturbances that are frequently observed in the postoperative patients. The clinical diagnosis is often empirical, thus requires a high index of suspicion. Further, the administration of corticosteroid replacement can be lifesaving. We report here a patient with acute adrenal crisis, who presented with shock immediately after surgical treatment of metastatic bone tumor of lung cancer origin.

## Case presentation

In April 2006, a 75-year old man who had been successfully treated for colon cancer 13 years ago, thyroid cancer 12 years ago, and was receiving endocrine therapy for prostate cancer from last year was transferred to our hospital for a fracture of his left proximal femur. The chest radiograph showed a solid mass in the hilum of left lung, and the thallium scintigram demonstrated abnormal uptake at the left proximal thigh and the hilum of left lung. In addition, the screening of tumor markers revealed extremely high level of CEA (1250 ng/ml), slightly high level of NSE (16.6 ng/ml) and SCC (2.2 ng/ml), and low level of thyroglobulin (0.3 ng/ml) and PSA (0.009 ng/ml). Thus the fracture was considered to be a bone metastasis from his fourth primary pulmonary cancer. Physical examination showed no hyperpigmentation of his all body surface. Laboratory investigation revealed serum sodium of 134 mmol/l, potassium 4.7 mmol/l, and plasma glucose 110 mg/dl, with normal renal and liver function.

He uneventfully underwent local excision and proximal femur replacement with a mega-prosthesis under general anesthesia. The bone tumor was pathologically diagnosed to be moderately differentiated adenocarcinoma. Later, the immunohistochemical examination revealed that cytokeratin 7 (CK7) and thyroid transcription factor 1 (TTF-1) were distinctly detected but cytokeratin 20 (CK20) never expressed. Recently, Chhieng et al. described that an adenocarcinoma was likely a primary lung tumor when it was of the CK7 positive/CK20 negative and TTF-1 positive phenotype [1]. Thus, the bone tumor was finally diagnosed to be a metastatic lung cancer.

**Figure 1:**
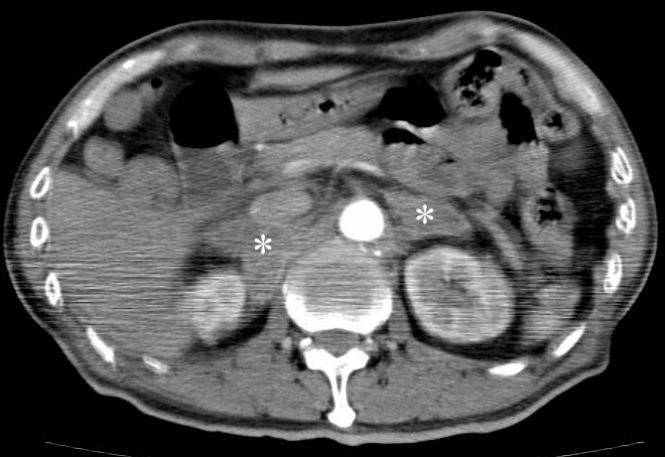
CT scan showing bilateral adrenal enlargement consistent with lung cancer metastases (asterisk).

The estimated intraoperative blood loss was 400 ml. The patient was extubated as usual, but the level of awakening was very poor. Postoperative laboratory examination demonstrated anemia and severe hyponatremia (126 mmol/l), but showed normokalemia (4.3 mmol/l) and normoglycemia (92 mg/dl). Despite the transfusion of blood and the administration of normal saline, the patient subsequently became hypotensive (SBP 60–70 mmHg), tachycardic, hypoxic, febrile (40.5°C) and confused. Re-intubation was required for respiratory distress. A chest radiograph showed diffuse pulmonary edema. We initially suspected this condition of acute pulmonary emboli. Immediately, the patient underwent CT examination from head to abdomen. Enhanced chest CT revealed a left hilar mass suggesting a primary lung cancer but no pulmonary embolus. A ventilation/perfusion scan performed on the next day showed no perfusion defect and confirmed definitely no evidence of pulmonary embolism. Concomitant brain and abdominal CT scan also demonstrated a low density area in the parietal lobe of the left brain and massively enlarged bilateral adrenal glands consistent with metastases (Figure 1). After the CT evaluation, we finally diagnosed adrenal crisis due to extensive destruction of adrenal tissue caused by metastases. Blood was drawn at 8:00 on the first postoperative day for serum cortisol levels that were found to be 2.0 μg/dl on the third postoperative day. Betamethasone (2 mg) was given to the patient at first, and switched to hydrocortisone, 100 mg administered intravenously every 8 hours. Dramatic improvement occurred in the subsequent hours following administration of hydrocortisone. The fever and hypotension promptly subsided and hyponatremia instantly disappeared. The patient recovered his consciousness and could be successfully extubated. On postoperative day 3, he was started on early remobilization by physical therapy. On postoperative day 7, the patient could sit down on the edge of a bed without assistance, followed by transfer exercise to a wheelchair. Although he could not undergo a CT-guided biopsy of the adrenal gland due to the flat refusal of his family, the following CT examination on postoperative day 35 demonstrated that bilateral adrenal glands neither decreased in size nor showed atrophic change, suggesting that adrenal enlargement was mainly caused by metastasis rather than hemorrhagic complication. The patient was transferred to the related hospital for further rehabilitation on postoperative day 39.

## Discussion

Acute adrenal crisis in the immediate post operative period is an uncommon problem. The clinical manifestations of this condition are not specific [2,3]. The subtle nature of symptoms, such as fever, hypotension, tachycardia, or confusion mimic the other more common postoperative complications and compound the difficulty in establishing the correct diagnosis. If the diagnosis is made in time adrenal crisis is managed easily, whereas if unrecognized this condition may cause serious morbidity and can be fatal. Therefore, the diagnosis of adrenal crisis should be considered in patients who become febrile, hypotensive, or confused after an operation despite adequate fluid replacement.

Additionally, a laboratory finding of severe hyponatremia and abdominal CT finding of bilateral adrenal enlargement provide important diagnostic clues to adrenal crisis in our patient, although the accurate serum cortisol measurement could not be obtained in the initial situation. Many threshold levels have been proposed for the definition of an insufficient cortisol level measured at any time [4]. Although none is entirely satisfactory [5], Cooper et al. stated that adrenal insufficiency appears to be likely when a random cortisol measurement is below 15 μg/dl during acute severe illness [6]. A more detailed work up such as the short corticotropin stimulation test would be desirable to confirm the diagnosis of primary adrenal insufficiency. However, Oelkers also described that morning plasma cortisol concentrations of ≤ 3 μg/dl are indicative of adrenal insufficiency and obviate the need for other tests [7]. Dynamic testing was not performed in our patient, because the serum cortisol level measured at the morning on the following day after operation was 2 μg/dl and clearly low. Furthermore, the prompt and dramatic response of steroid replacement strongly supported the diagnosis of adrenal crisis.

Adrenal crisis during the course of an unstable period after orthopedic joint surgery is rarely reported. Review of the literature reveals only four case reports (Table 1) [8-11]. All of them were free from malignancy. They underwent total knee arthroplasty for severe osteoarthritis and received anticoagulant agents (heparin and/or warfarin) postoperatively. Several days after surgery, symptoms due to adrenal insufficiency occurred. Among them, two patients were successfully treated under the correct diagnosis, whereas the other two died without appropriate treatment and postmortem examination demonstrated severe bilateral adrenal enlargement and hemorrhagic change. The authors argued that acute adrenal crisis as a consequence of bilateral adrenal hemorrhage should be considered in the differential diagnosis of patients who show medical deterioration and instability after having undergone surgical procedures such as joint replacement, especially when anticoagulant agents have been utilized for prophylaxis against deep vein thrombosis.

**Table 1:** Case reports of adrenal crisis after orthopedic joint surgery

Authors	Gender*/age	preceding surgery^†^	anticoagulant agent used	beginning of the symptoms (postoperative day)	prognosis
Ries et al [8]	F/61	bil. TKA	warfarin	day6	dead
Cozzolino et al [9]	F/66	rt. TKA	warfarin	shortly after discharge from hospital	alive
LaBan et al [10]	F/82	bil. TKA	heparin followed by warfarin	day8	alive
Schuchmann et al [11]	F/83	bil. TKA	heparin followed by enoxaparin	day5	dead

*F, female

^†^bil., bilateral; rt., right; TKA, total knee arthroplasty

The immunohistochemical results strongly suggested that the origin of bone tumor in this case might be a lung cancer. In addition to bone, the brain and adrenal glands are known to be common sites of metastatic spread from lung cancer. However, due to the physiologic accumulation to the liver and kidney, metastatic adrenal cancer could not be detected by the preoperative thallium scintigram showing abnormal uptake at the lung and femur. The emergent CT exhibiting massively enlarged bilateral adrenal gland and the following CT showing no decrease in size of adrenal glands implied that adrenal enlargement was due to metastases to both adrenal glands, although adrenal hemorrhage could show normal or slight high intensity on CT as this case [12,13]. In the present case, anticoagulant agents were not used and symptoms took place immediately after surgery different from the cases with adrenal hemorrhage. Taken together, adrenal crisis in this case was considered to be caused by the lack of adrenal reserve based on metastatic involvement and surgical stress.

Patients with pathologic fracture of a weight bearing limb should be treated surgically because of severe pain, gait disability and decreases in activities of daily living (ADL) and quality of life (QOL). Recent advances in cancer treatment cause the prolonged survival and increase the prevalence of bone metastasis. Consequently, orthopedic surgeons have been further involved in the diagnosis and treatment of the metastatic bone tumors. To our best knowledge, there have been no reports of adrenal crisis after the surgery for pathologic fracture. However, adrenal glands are commonly involved by metastatic disease from various neoplasms including lung cancer. Besides, prospective study has found that up to one third of patients found to have bilateral adrenal metastases secondary to neoplastic disease will ultimately develop adrenal crisis [14]. Adrenal crisis may be overlooked among patients with pathologic fractures. Thus, we recommend that visualization of the adrenals using abdominal CT should be considered in the work up of pathologic fractures. In patients with bilateral adrenal enlargement, screening for adrenal insufficiency with a basal hormone measurement and a corticotropin stimulation test may be indicated. We hope that this case report will alert surgeons to the possibility of postoperative adrenal crisis in patients with bone metastases, especially from lung cancer.

## Conclusion

We describe a case of adrenal crisis caused by the lack of adrenal reserve based on metastatic involvement and surgical stress, the first published case of adrenal crisis after surgery for a pathologic fracture from lung cancer metastasis. Surgeons treating pathologic fractures should be aware of this complication and familiar with its appropriate therapy because of increasing opportunity to care patients with metastatic bone tumors due to recent advances in cancer treatment.

## List of Abbreviations used

computed tomography, CT; magnetic resonance imaging, MRI.

## Conflict of interests

The author(s) declare that they have no competing interests.

## Authors' contributions

**NN **wrote the case report and performed the literature search

**ST **performed the operation and helped in preparing the manuscript

**KN **performed the operation and helped in preparing the manuscript

**YM **performed the operation and helped in preparing the manuscript

**BC **participated in managing the case as an anesthesiologist

**SS **participated in managing the case as an anesthesiologist

**NH **performed the literature search and helped to draft the manuscript

**YT **performed the pathological diagnosis

**NA **revised the manuscript

All authors read and approved the final manuscript.
